# Isomorphous crystal structures of chlorodi­acetyl­ene and iododi­acetyl­ene derivatives: simultaneous hydrogen and halogen bonds on carbon­yl

**DOI:** 10.1107/S2056989017010155

**Published:** 2017-07-17

**Authors:** Pierre Baillargeon, Tarik Rahem, Édouard Caron-Duval, Jacob Tremblay, Cloé Fortin, Étienne Blais, Victor Fan, Daniel Fortin, Yves L. Dory

**Affiliations:** a Département de Chimie, Cégep de Sherbrooke, 475 rue du Cégep, Sherbrooke, Québec, J1E 4K1, Canada; b Laboratoire d’Analyses Structurales par Diffraction des Rayons-X, Département de Chimie, Université de Sherbrooke, 2500, boulevard de l’Université, Sherbrooke, Québec, J1K 2R1, Canada; c Laboratoire de Synthèse Supramoléculaire, Département de Chimie, Institut de Pharmacologie, Université de Sherbrooke, 3001 12e avenue nord, Sherbrooke, QC, J1H 5N4, Canada

**Keywords:** crystal structure, halogen bond, hydrogen bond, chlorodi­acetyl­ene, iododi­acetyl­ene

## Abstract

The crystal structures of *tert*-butyl (5-chloro­penta-2,4-diyn-1-yl)carbamate, C_10_H_12_ClNO_2_ (**II**), and *tert*-butyl (5-iodo­penta-2,4-diyn-1-yl)carbamate, C_10_H_12_INO_2_ (**IV**), are isomorphous to previously reported structures and accordingly their mol­ecular and supra­molecular structures are similar. In the crystals of (**II**) and (**IV**), mol­ecules are linked into very similar two-dimensional wall organizations with anti­parallel carbamate groups involved in a combination of hydrogen and halogen bonds (bifurcated N—H⋯O=C and C≡C—*X*⋯O=C inter­actions on the same carbonyl group). There is no long-range parallel stacking of diynes, so the topochemical polymerization of di­acetyl­ene is prevented. A Cambridge Structural Database search revealed that C≡C—*X*⋯O=C contacts shorter than the sum of the van der Waals radii are scarce (only one structure for the C≡C—Cl⋯O=C inter­action and 13 structures for the similar C≡C—I⋯O=C inter­action).

## Abstract 2

*tert*-Butyl (5-chloro­penta-2,4-diyn-1-yl)carbamate and *tert*-butyl (5-iodo­penta-2,4-diyn-1-yl)carbamate are new members of the isostructural family of compounds with the general formula BocNHCH_2_CCCC*X* (*X* = H, Cl, Br, I). In the crystals of all these di­acetyl­enes, mol­ecules are linked *via* a bifurcated N—H⋯O hydrogen bond and C—*X*⋯O halogen bond involving the same carbonyl group.

## Chemical context   

Hydrogen bonds (HBs) and halogen bonds (XBs) are considered to be useful noncovalent synthetic tools in crystal engineering (Aakeröy *et al.*, 2015 ▸; Grabowski, 2016 ▸; Resnati *et al.*, 2015 ▸; Cinčić *et al.*, 2008 ▸). Indeed, these directional inter­molecular inter­actions facilitate the preparation of the desired solid-state motifs and architectures (Gilday *et al.*, 2015 ▸; Cavallo *et al.*, 2016 ▸; Priimagi *et al.*, 2013 ▸; Mukherjee *et al.*, 2014 ▸; Shirman *et al.*, 2015 ▸; Mukherjee *et al.*, 2017 ▸). For example, using HBs and XBs, the specific organization of terminal di­acetyl­enes (Li *et al.*, 2009 ▸; Ouyang *et al.*, 2003 ▸), bromodi­acetyl­enes (Jin *et al.*, 2015 ▸) and iododi­acetyl­enes (Jin *et al.*, 2013 ▸; Sun *et al.*, 2006 ▸) has been obtained to achieve the solid-state topochemical polymerization of di­acetyl­enes. On the other hand, to the best of our knowledge, no chlorodi­acetyl­ene topochemical polymerizations have been reported. Our results show that chlorodi­acetyl­ene (**II**) is isostructural to iododi­acetyl­ene (**IV**) and the previously reported bromodi­acetyl­ene (**III**) and terminal di­acetyl­ene (**I**) (Baillargeon *et al.*, 2016 ▸) (see Scheme[Chem scheme1]). Although the arrangement of diynes in the present article stands no chance of undergoing topochemical polymerization, we suggest that in other systems prone to polymerization, replacing Br, I or H atoms by Cl atoms in their diyne groups might result in successful PolyChloroDi­Acetyl­ene (PCDA) formation as well. This work also contributes to an emerging research theme, namely the concept of orthogonal mol­ecular inter­actions such as HBs and XBs (Kratzer *et al.*, 2015 ▸; Takemura *et al.*, 2014 ▸; Voth *et al.*, 2009 ▸), which may find applications in medicinal chemistry and chemical biology (Wilcken *et al.*, 2013 ▸).
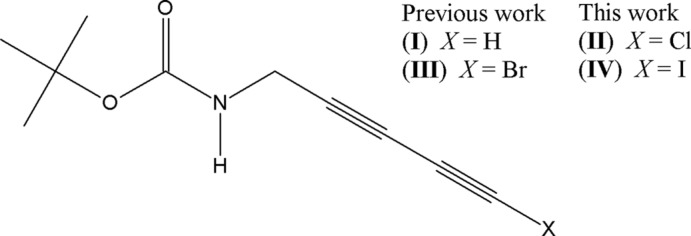


## Structural commentary   

**Figure 1:**
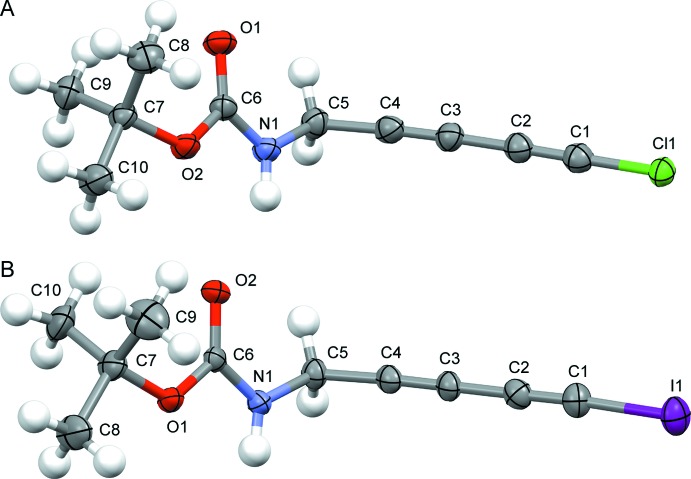
The mol­ecular structure of (A) compound (**II**) and (B) compound (**IV**), showing the atom-labelling schemes. Displacement ellipsoids are drawn at the 50% probability level. H atoms are shown as fixed-size spheres of 0.30 Å.

The mol­ecular structures of compounds (**II**) and (**IV**) are shown in Fig. 1 ▸. All bond lengths and angles are within normal ranges. For example, the inter­nal diyne C2—C3 bonds lengths [1.376 (3) Å for (**II**) and 1.385 (4) Å for (**IV**)] follow the useful rule of thumb describing a C—C single-bond distance (1.54 Å) decreasing by 0.04 Å each time one of the participating C atoms changes hybridization from *sp* ^3^ to *sp* ^2^ or from *sp* ^2^ to *sp* (Bent, 1961 ▸). Moreover, the observed distances are almost identical to those found recently in the literature for similar halodiynes (Hoheisel *et al.*, 2013 ▸; Baillargeon *et al.*, 2016 ▸). The relative orientation between the di­acetyl­enic moiety and the carbamate functional group can be established by the absolute value of the torsion angles C4—C5—N1—C6 [111.07 (19)°] for (**II**) and [103.8 (3)°] for (**IV**).

## Supra­molecular features   

**Figure 2:**
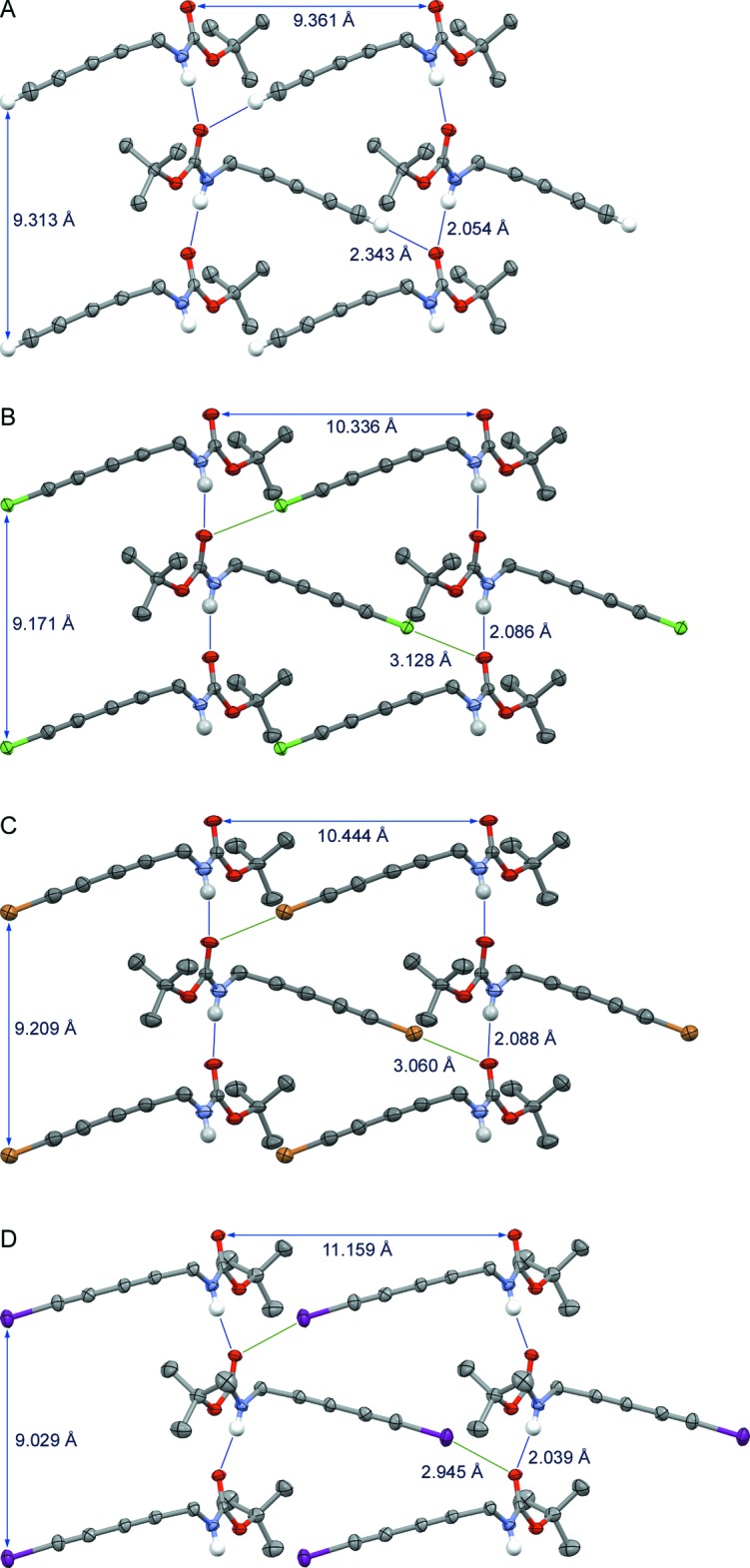
Halogen (green lines) and/or hydrogen bonds (blue lines) inside the supra­molecular walls of (A) diyne (**I**), (B) chloro­diyne (**II**), (C) bromo­diyne (**III**) and (D) iodo­diyne (**IV**). The nonpolar H atoms have been omitted for clarity.

In the crystals of compounds (**II**) and (**IV**), mol­ecules are linked *via* an N—H⋯O=C hydrogen bond between their respective carbamate functionalities [N1—H1⋯O1^i^ (Table 1 ▸) and N1—H1⋯O2^i^ (Table 2 ▸)], generating an anti­parallel stacking pattern which orients the di­acetyl­ene skeleton on each side of the one-dimensional carbamate tape (parts B and D in Fig. 2 ▸). For both crystals, the simultaneous presence of halogen and hydrogen bonds with the carbamate O atom have been found. Indeed, additional halogen-bond inter­actions occur with the carbamate O atom [Cl1⋯O1^ii^ for (**II**) and I1⋯O2^ii^ for (**IV**)], resulting in an infinite two-dimensional network that can be considered as polar supra­molecular walls. This arrangement is similar to our previous work (Baillargeon *et al.*, 2016 ▸) on the terminal di­acetyl­ene (**I**) (part A in Fig. 2 ▸) and the bromodi­acetyl­ene (**III**) (part C in Fig. 2 ▸). In fact, diynes (**I**)–(**IV**) (Fig. 2 ▸) constitute a complete set of truly isomorphous crystals that can be carefully examined to evaluate the differences and similarities that exist between halogen and hydrogen bonds. Thus, the *X*⋯O⋯H angle increases as the size of the halogen atom becomes larger. This angle, which is pretty open in the chlorine crystal (**II**) (Cl1⋯O1⋯H1; part B in Fig. 2 ▸; 69°) adopts a near orthogonal geometry with the iodine (I1⋯O2⋯H1; part D in Fig. 2 ▸; 83°). It is not a surprise that the bromine crystal (**III**) represents an inter­mediate case (part C in Fig. 2 ▸; 72°). The value for the terminal di­acetyl­ene (**I**) *X* = H (part A in Fig. 2 ▸; 76°) is closely related to the bromodi­acetyl­ene (Baillargeon *et al.*, 2016 ▸).

## Database survey   

A survey of the Cambridge Structural Database (*Conquest* Version 1.19; CSD, Version 5.38, November 2016 plus 3 updates; Groom *et al.*, 2016 ▸) furnished 404 hits of terminal alkynes CC—H having close contacts with carbonyl O=C (shorter than the sum of their van der Waals radii). On the other hand, similar contacts from halogenoalkyne analogs are scarce (1 hit for the chloro­alkyne, 4 hits for the bromo­alkyne and 13 hits for the iodo­alkyne; Table 3 ▸). For the iodo­alkyne, results are limited to monovalent iodine and for a structure in which the carbonyl group is not involved in an organometallic complex.

## Synthesis and crystallization   

### Compound (II)   

Tetra-*n*-butylammonium fluoride (TBAF, 0.437 ml, 1 *M* in THF, 0.437 mmol), AgNO_3_ (39 mg, 0.23 mmol) and NCS (190 mg, 1.42 mmol) were added to a solution of BocNHCH_2_—C≡C—C≡C—TMS (183 mg, 0.728 mmol) in aceto­nitrile (3 ml) at room temperature. The resulting mixture was stirred for 2.5 h under N_2_ in the absence of light. Purification of the crude product by flash chromatography on silica gel, eluting with mixtures of Hex/DCM/Et_2_O (gradient from 9:1:1 to 1:1:1), provided compound **(II)** as a beige solid (yield 72 mg, 46%). Single crystals suitable for X-ray diffraction were prepared by diffusion of pentane into a chloro­form solution of (**II**) at 263 K. *R*_F_ = 0.43 (2:1:1 Hex/DCM/Et_2_O); IR (UATR, ν, cm^−1^): 3326, 2977, 2920, 2255, 2168, 1673, 1531, 1421, 1368, 1278, 1248, 1222, 1158, 1143, 1042, 1028, 933, 849, 761, 718, 655; ^1^H NMR (400 MHz, CDCl_3_): δ 4.72 (*br*, 1H), 3.99 (*d*, 2H), 1.45 (*s*, 9H); HRMS (*m*/*z*): calculated for C_10_H_12_ClNNaO_2_ [*M*Na^+^]: 236.0449, found: 236.0448.

### Compound (IV)   

TBAF (0.437 ml, 1 *M* in THF, 0.437 mmol), AgNO_3_ (39 mg, 0.23 mmol) and NIS (328 mg, 1.46 mmol) were added to a solution of BocNHCH_2_—C≡C—C≡C—TMS (183 mg, 0.728 mmol) in aceto­nitrile (3 ml) at room temperature. The resulting mixture was stirred for 2.5 h under N_2_ in the absence of light. Purification of the crude product by flash chromatography on silica gel, eluting with mixtures of Hex/DCM/Et_2_O (gradient from 9:1:1 to 1:1:1) provided compound **(IV)** as a beige solid (yield 95 mg, 43%). Single crystals suitable for X-ray diffraction were prepared by slow evaporation from a chloro­form solution of (**IV**) at room temperature. *R*_F_ = 0.48 (2:1:1 Hex/DCM/Et_2_O); IR (UATR, ν, cm^−1^): 3328, 2980, 2933, 2230, 2159, 1661, 1532, 1451, 1420, 1367, 1284, 1250, 1154, 1142, 1042, 1026, 929, 851, 762, 714, 647; ^1^H NMR (400 MHz, CDCl_3_): δ 4.73 (*br*, 1H), 4.02 (*d*, 2H), 1.44 (*s*, 9H).

## Refinement   

Crystal data, data collection and structure refinement details are summarized in Table 4 ▸.

## Supplementary Material

Crystal structure: contains datablock(s) global, II, IV. DOI: 10.1107/S2056989017010155/mw2133sup1.cif

Structure factors: contains datablock(s) II. DOI: 10.1107/S2056989017010155/mw2133IIsup2.hkl

Structure factors: contains datablock(s) IV. DOI: 10.1107/S2056989017010155/mw2133IVsup3.hkl

CCDC references: 1551031, 1551032

Additional supporting information:  crystallographic information; 3D view; checkCIF report

## Figures and Tables

**Table 1:** Hydrogen-bond and halogen-bond geometries (Å, °) for (**II**)

*D*—*X*⋯*A*	*D*—*X*	*X*⋯*A*	*D*⋯*A*	*D*—*X*⋯*A*
N1—H1⋯O1^i^	0.88	2.09	2.934 (2)	162
C1—Cl1⋯O1^ii^	1.665 (2)	3.127 (2)	4.792 (3)	179.01 (7)

Symmetry codes: (i) 
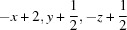
; (ii) 
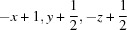
.

**Table 2:** Hydrogen-bond and halogen-bond geometries (Å, °) for (**IV**)

*D*—*X*⋯*A*	*D*—*X*	*X*⋯*A*	*D*⋯*A*	*D*—*X*⋯*A*
N1—H1⋯O2^i^	0.88	2.04	2.881 (2)	160
C1—I1⋯O2^ii^	1.999 (2)	2.945 (2)	4.919 (3)	168.31 (8)

Symmetry codes: (i) 
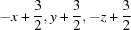
; (ii) 
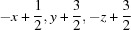
.

**Table 3:** CSD data (Groom *et al.*, 2016 ▸) retrieved for the C≡C—*X*⋯O=C contacts shorter than the sum of their van der Waals radii

C≡C—*X*⋯O=C contacts	CSD refcode	Space group	*X*⋯O distance (Å)	C—*X*⋯O angle (°)	Reference
C≡C—Cl⋯O=C	NIDWAA	*P* 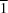	3.111; 3.241	152.59; 158.76	Kawai *et al.* (2013 ▸)
C≡C—Br⋯O=C	HEVWAI	*C*2	2.959	158.12	Hoheisel *et al.* (2013 ▸)
C≡C—Br⋯O=C	HEVWAI01	*P*2_1_2_1_2_1_	2.966	166.70	Hoheisel *et al.* (2013 ▸)
C≡C—Br⋯O=C	NIDWII	*P*2_1_/*n*	2.867	171.11	Kawai *et al.* (2013 ▸)
C≡C—Br⋯O=C	KAMXII	*P*2_1_/*c*	3.060	178.26	Baillargeon *et al.* (2016 ▸)
C≡C—I⋯O=C	COHYUU	*P* 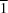	3.096	164.55	Luo *et al.* (2008 ▸)
C≡C—I⋯O=C	IYAYUC	*Pca*2_1_	2.861	170.36	Hou *et al.* (2004 ▸)
C≡C—I⋯O=C	MASVUZ	*P*2_1_/*n*	2.834; 2.887	170.72; 172.97	Perkins *et al.* (2012 ▸)
C≡C—I⋯O=C	TOYPUS	*P*2_1_/*c*	2.933	175.36	Avtomonov *et al.* (1997 ▸)
C≡C—I⋯O=C	HOWXIC	*P*2_1_/*c*	2.887	169.51	Dumele *et al.* (2014 ▸)
C≡C—I⋯O=C	LUNKOW	*P*2/*c*	2.791	174.12	Kratzer *et al.* (2015 ▸)
C≡C—I⋯O=C	LUNKUC	*P*2_1_/*c*	2.754	172.63	Kratzer *et al.* (2015 ▸)
C≡C—I⋯O=C	LUNLAJ	*P*2_1_/*c*	2.773	173.70	Kratzer *et al.* (2015 ▸)
C≡C—I⋯O=C	LUNLIR	*Pca*2_1_	2.858	170.94	Kratzer *et al.* (2015 ▸)
C≡C—I⋯O=C	LUNLOX	*C*2/*c*	2.763	175.58	Kratzer *et al.* (2015 ▸)
C≡C—I⋯O=C	IBUYAI	*P*2_1_/*m*	2.856	177.96	Dumele *et al.* (2017 ▸)
C≡C—I⋯O=C	IBUYOW	*P*2_1_/*c*	2.830	176.52	Dumele *et al.* (2017 ▸)
C≡C—I⋯O=C	IBUYUC	*P* 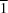	2.878	177.89	Dumele *et al.* (2017 ▸)

**Table 4:** Experimental details

	(**II**)	(**IV**)
Crystal data		
Chemical formula	C_10_H_12_ClNO_2_	C_10_H_12_INO_2_
*M*_r_	213.66	305.11
Crystal system, space group	Monoclinic, *P*2_1_/*c*	Monoclinic, *P*2_1_/*n*
Temperature (K)	173	173
*a*, *b*, *c* (Å)	10.336 (3), 9.171 (3), 11.870 (3)	11.1587 (16), 9.0288 (13), 12.9899 (18)
β (°)	100.656 (5)	108.731 (2)
*V* (Å^3^)	1105.8 (5)	1239.4 (3)
*Z*	4	4
Radiation type	Mo *K*α	Mo *K*α
μ (mm^−1^)	0.32	2.56
Crystal size (mm)	0.34 × 0.22 × 0.02	0.36 × 0.3 × 0.28

Data collection		
Diffractometer	Bruker APEXII	Bruker APEXII
Absorption correction	Multi-scan (*SADABS*; Bruker, 2008 ▸)	Multi-scan (*SADABS*; Bruker, 2008 ▸)
*T*_min_, *T*_max_	0.66, 0.745	0.675, 0.745
No. of measured, independent and observed [*I* > 2σ(*I*)] reflections	16132, 2249, 1755	17970, 2532, 2342
*R*_int_	0.045	0.02
(sin θ/λ)_max_ (Å^−1^)	0.625	0.626

Refinement		
*R*[*F* ^2^ > 2σ(*F* ^2^)], *wR*(*F* ^2^), *S*	0.036, 0.089, 1.06	0.022, 0.054, 1.08
No. of reflections	2249	2532
No. of parameters	130	130
H-atom treatment	H-atom parameters constrained	H-atom parameters constrained
Δρ_max_, Δρ_min_ (e Å^−3^)	0.22, −0.21	1.32, −0.69

Computer programs: *APEX2* (Bruker, 2008 ▸), *SAINT* (Bruker, 2008 ▸), *SORTAV* (Blessing, 1995 ▸), *SHELXS97* (Sheldrick, 2008 ▸), *SHELXL2016* (Sheldrick, 2015 ▸), *ORTEP-3 for Windows* (Farrugia, 2012 ▸), *Mercury* (Macrae *et al.*, 2006 ▸), *WinGX* (Farrugia, 2012 ▸) and *publCIF* (Westrip, 2010 ▸).

## supplementary crystallographic information

### tert-Butyl (5-chloropenta-2,4-diyn-1-yl)carbamate (II) Crystal data

**Table:**

C_10_H_12_ClNO_2_	*F*(000) = 448
*M**_r_* = 213.66	*D*_x_ = 1.283 Mg m^−^^3^
Monoclinic, *P*2_1_/*c*	Mo *K*α radiation, λ = 0.71073 Å
Hall symbol: -P 2ybc	Cell parameters from 8841 reflections
*a* = 10.336 (3) Å	θ = 2.8–26.4°
*b* = 9.171 (3) Å	µ = 0.32 mm^−^^1^
*c* = 11.870 (3) Å	*T* = 173 K
β = 100.656 (5)°	Plate, orange
*V* = 1105.8 (5) Å^3^	0.34 × 0.22 × 0.02 mm
*Z* = 4	

### tert-Butyl (5-chloropenta-2,4-diyn-1-yl)carbamate (II) Data collection

**Table:**

Bruker APEXII diffractometer	2249 independent reflections
Radiation source: sealed x-ray tube	1755 reflections with *I* > 2σ(*I*)
Graphite monochromator	*R*_int_ = 0.045
φ or ω oscillation scans	θ_max_ = 26.4°, θ_min_ = 2.0°
Absorption correction: multi-scan (SADABS; Bruker, 2008)	*h* = −12→12
*T*_min_ = 0.66, *T*_max_ = 0.745	*k* = −11→11
16132 measured reflections	*l* = −9→14

### tert-Butyl (5-chloropenta-2,4-diyn-1-yl)carbamate (II) Refinement

**Table:**

Refinement on *F*^2^	0 constraints
Least-squares matrix: full	Hydrogen site location: inferred from neighbouring sites
*R*[*F*^2^ > 2σ(*F*^2^)] = 0.036	H-atom parameters constrained
*wR*(*F*^2^) = 0.089	*w* = 1/[σ^2^(*F*_o_^2^) + (0.0397*P*)^2^ + 0.2863*P*] where *P* = (*F*_o_^2^ + 2*F*_c_^2^)/3
*S* = 1.06	(Δ/σ)_max_ < 0.001
2249 reflections	Δρ_max_ = 0.22 e Å^−^^3^
130 parameters	Δρ_min_ = −0.21 e Å^−^^3^
0 restraints	

### tert-Butyl (5-chloropenta-2,4-diyn-1-yl)carbamate (II) Special details

**Table:**

Geometry. All esds (except the esd in the dihedral angle between two l.s. planes) are estimated using the full covariance matrix. The cell esds are taken into account individually in the estimation of esds in distances, angles and torsion angles; correlations between esds in cell parameters are only used when they are defined by crystal symmetry. An approximate (isotropic) treatment of cell esds is used for estimating esds involving l.s. planes.

### tert-Butyl (5-chloropenta-2,4-diyn-1-yl)carbamate (II) Fractional atomic coordinates and isotropic or equivalent isotropic displacement parameters (Å^2^)

**Table:**

	*x*	*y*	*z*	*U*_iso_*/*U*_eq_	
C1	0.40035 (19)	0.0211 (2)	0.15762 (16)	0.0343 (4)	
C2	0.50716 (18)	−0.0266 (2)	0.15615 (16)	0.0336 (4)	
C3	0.63096 (19)	−0.0798 (2)	0.15309 (16)	0.0336 (4)	
C4	0.73990 (19)	−0.1223 (2)	0.15037 (16)	0.0334 (4)	
C5	0.87519 (17)	−0.1687 (2)	0.14761 (17)	0.0339 (4)	
H5A	0.882935	−0.274828	0.16243	0.041*	
H5B	0.894345	−0.150569	0.070125	0.041*	
C6	1.04144 (17)	−0.15939 (19)	0.32333 (15)	0.0271 (4)	
C7	1.22986 (18)	−0.11065 (19)	0.47967 (15)	0.0304 (4)	
C8	1.1748 (2)	−0.1841 (2)	0.57483 (17)	0.0439 (5)	
H8A	1.106364	−0.122368	0.597047	0.066*	
H8B	1.245644	−0.198955	0.641132	0.066*	
H8C	1.136913	−0.278618	0.547923	0.066*	
C9	1.32333 (19)	−0.2075 (2)	0.42986 (18)	0.0408 (5)	
H9A	1.278745	−0.298815	0.40299	0.061*	
H9B	1.400577	−0.228971	0.488902	0.061*	
H9C	1.351283	−0.15766	0.365371	0.061*	
C10	1.2953 (2)	0.0332 (2)	0.52078 (19)	0.0449 (5)	
H10A	1.324136	0.082715	0.456527	0.067*	
H10B	1.371735	0.014414	0.581378	0.067*	
H10C	1.232365	0.095115	0.550916	0.067*	
Cl1	0.24981 (4)	0.08590 (5)	0.15756 (4)	0.03061 (14)	
N1	0.97160 (14)	−0.09282 (16)	0.23144 (13)	0.0314 (4)	
H1	0.984766	0.000739	0.221551	0.038*	
O1	1.03225 (13)	−0.28874 (13)	0.34594 (11)	0.0361 (3)	
O2	1.12149 (12)	−0.06256 (12)	0.38708 (11)	0.0311 (3)	

### tert-Butyl (5-chloropenta-2,4-diyn-1-yl)carbamate (II) Atomic displacement parameters (Å^2^)

**Table:**

	*U*^11^	*U*^22^	*U*^33^	*U*^12^	*U*^13^	*U*^23^
C1	0.0342 (11)	0.0349 (11)	0.0334 (11)	−0.0009 (9)	0.0055 (8)	0.0009 (8)
C2	0.0337 (11)	0.0333 (10)	0.0323 (11)	−0.0036 (8)	0.0019 (8)	0.0007 (8)
C3	0.0333 (11)	0.0300 (10)	0.0346 (11)	−0.0005 (8)	−0.0011 (8)	0.0016 (8)
C4	0.0339 (11)	0.0279 (10)	0.0350 (11)	−0.0023 (8)	−0.0028 (8)	−0.0006 (8)
C5	0.0309 (10)	0.0311 (10)	0.0368 (11)	0.0013 (8)	−0.0014 (8)	−0.0030 (8)
C6	0.0264 (9)	0.0222 (9)	0.0323 (10)	−0.0014 (7)	0.0045 (7)	−0.0023 (7)
C7	0.0311 (10)	0.0269 (10)	0.0299 (10)	0.0017 (8)	−0.0026 (8)	0.0021 (8)
C8	0.0524 (13)	0.0429 (12)	0.0369 (12)	0.0014 (10)	0.0091 (10)	0.0049 (9)
C9	0.0344 (11)	0.0415 (12)	0.0451 (13)	0.0056 (9)	0.0037 (9)	0.0019 (9)
C10	0.0468 (13)	0.0339 (11)	0.0462 (13)	−0.0041 (10)	−0.0121 (10)	−0.0007 (9)
Cl1	0.0268 (2)	0.0320 (3)	0.0337 (3)	0.00451 (19)	0.00733 (18)	0.00090 (19)
N1	0.0303 (8)	0.0220 (8)	0.0381 (9)	−0.0024 (7)	−0.0036 (7)	0.0013 (7)
O1	0.0409 (8)	0.0217 (7)	0.0427 (8)	−0.0034 (6)	0.0003 (6)	0.0014 (6)
O2	0.0314 (7)	0.0220 (7)	0.0358 (8)	−0.0009 (5)	−0.0049 (6)	−0.0006 (5)

### tert-Butyl (5-chloropenta-2,4-diyn-1-yl)carbamate (II) Geometric parameters (Å, º)

**Table:**

C1—C2	1.191 (3)	C6—N1	1.339 (2)
C1—Cl1	1.666 (2)	C6—O2	1.347 (2)
C2—C3	1.376 (3)	C7—O2	1.484 (2)
C3—C4	1.198 (3)	C7—C9	1.511 (3)
C4—C5	1.468 (3)	C7—C8	1.513 (3)
C5—N1	1.449 (2)	C7—C10	1.521 (3)
C6—O1	1.224 (2)		
			
C2—C1—Cl1	178.9 (2)	O2—C7—C9	109.55 (15)
C1—C2—C3	179.0 (2)	O2—C7—C8	110.41 (15)
C4—C3—C2	178.2 (2)	C9—C7—C8	112.70 (16)
C3—C4—C5	177.8 (2)	O2—C7—C10	102.12 (14)
N1—C5—C4	112.49 (16)	C9—C7—C10	110.89 (17)
O1—C6—N1	124.66 (17)	C8—C7—C10	110.67 (17)
O1—C6—O2	125.48 (17)	C6—N1—C5	122.64 (16)
N1—C6—O2	109.86 (15)	C6—O2—C7	121.42 (13)
			
O1—C6—N1—C5	0.3 (3)	N1—C6—O2—C7	−166.78 (14)
O2—C6—N1—C5	−178.98 (15)	C9—C7—O2—C6	59.3 (2)
C4—C5—N1—C6	111.1 (2)	C8—C7—O2—C6	−65.4 (2)
O1—C6—O2—C7	13.9 (3)	C10—C7—O2—C6	176.85 (16)

### tert-Butyl (5-chloropenta-2,4-diyn-1-yl)carbamate (II) Hydrogen-bond geometry (Å, º)

**Table:**

*D*—H···*A*	*D*—H	H···*A*	*D*···*A*	*D*—H···*A*
N1—H1···O1^i^	0.88	2.09	2.935	162
C1—Cl1···O1^ii^	1.67	3.13	4.793	179

Symmetry codes: (i) −*x*+2, *y*+1/2, −*z*+1/2; (ii) −*x*+1, *y*+1/2, −*z*+1/2.

### tert-Butyl (5-iodopenta-2,4-diyn-1-yl)carbamate (IV) Crystal data

**Table:**

C_10_H_12_INO_2_	*F*(000) = 592
*M**_r_* = 305.11	*D*_x_ = 1.635 Mg m^−^^3^
Monoclinic, *P*2_1_/*n*	Mo *K*α radiation, λ = 0.71073 Å
Hall symbol: -P 2yn	Cell parameters from 9940 reflections
*a* = 11.1587 (16) Å	θ = 2.3–26.4°
*b* = 9.0288 (13) Å	µ = 2.56 mm^−^^1^
*c* = 12.9899 (18) Å	*T* = 173 K
β = 108.731 (2)°	Prism, yellow
*V* = 1239.4 (3) Å^3^	0.36 × 0.3 × 0.28 mm
*Z* = 4	

### tert-Butyl (5-iodopenta-2,4-diyn-1-yl)carbamate (IV) Data collection

**Table:**

Bruker APEXII diffractometer	2532 independent reflections
Radiation source: sealed x-ray tube	2342 reflections with *I* > 2σ(*I*)
Graphite monochromator	*R*_int_ = 0.02
φ or ω oscillation scans	θ_max_ = 26.4°, θ_min_ = 2.1°
Absorption correction: multi-scan (SADABS; Bruker, 2008)	*h* = −12→13
*T*_min_ = 0.675, *T*_max_ = 0.745	*k* = −11→11
17970 measured reflections	*l* = −15→16

### tert-Butyl (5-iodopenta-2,4-diyn-1-yl)carbamate (IV) Refinement

**Table:**

Refinement on *F*^2^	0 constraints
Least-squares matrix: full	Hydrogen site location: inferred from neighbouring sites
*R*[*F*^2^ > 2σ(*F*^2^)] = 0.022	H-atom parameters constrained
*wR*(*F*^2^) = 0.054	*w* = 1/[σ^2^(*F*_o_^2^) + (0.0203*P*)^2^ + 1.7447*P*] where *P* = (*F*_o_^2^ + 2*F*_c_^2^)/3
*S* = 1.08	(Δ/σ)_max_ = 0.001
2532 reflections	Δρ_max_ = 1.32 e Å^−^^3^
130 parameters	Δρ_min_ = −0.69 e Å^−^^3^
0 restraints	

### tert-Butyl (5-iodopenta-2,4-diyn-1-yl)carbamate (IV) Special details

**Table:**

Geometry. All esds (except the esd in the dihedral angle between two l.s. planes) are estimated using the full covariance matrix. The cell esds are taken into account individually in the estimation of esds in distances, angles and torsion angles; correlations between esds in cell parameters are only used when they are defined by crystal symmetry. An approximate (isotropic) treatment of cell esds is used for estimating esds involving l.s. planes.

### tert-Butyl (5-iodopenta-2,4-diyn-1-yl)carbamate (IV) Fractional atomic coordinates and isotropic or equivalent isotropic displacement parameters (Å^2^)

**Table:**

	*x*	*y*	*z*	*U*_iso_*/*U*_eq_	
C1	0.2157 (2)	1.0340 (3)	0.3437 (2)	0.0328 (6)	
C2	0.3210 (2)	1.0012 (3)	0.34873 (19)	0.0304 (5)	
C3	0.4425 (2)	0.9623 (3)	0.35280 (19)	0.0279 (5)	
C4	0.5465 (2)	0.9286 (3)	0.35525 (19)	0.0275 (5)	
C5	0.6760 (2)	0.8938 (3)	0.35692 (19)	0.0282 (5)	
H5A	0.736704	0.927089	0.42691	0.034*	
H5B	0.684833	0.785076	0.352172	0.034*	
C6	0.7111 (2)	0.8882 (2)	0.18051 (19)	0.0219 (4)	
C7	0.7579 (3)	0.9226 (3)	0.0094 (2)	0.0344 (6)	
C8	0.7976 (4)	1.0622 (3)	−0.0367 (3)	0.0511 (8)	
H8A	0.730469	1.136625	−0.049832	0.077*	
H8B	0.812386	1.038609	−0.105307	0.077*	
H8C	0.875616	1.101212	0.015278	0.077*	
C9	0.6338 (4)	0.8624 (4)	−0.0654 (3)	0.0559 (9)	
H9A	0.611252	0.772368	−0.033698	0.084*	
H9B	0.642508	0.839113	−0.136337	0.084*	
H9C	0.567195	0.936832	−0.074587	0.084*	
C10	0.8637 (3)	0.8100 (4)	0.0377 (3)	0.0506 (8)	
H10A	0.936443	0.849222	0.095807	0.076*	
H10B	0.888703	0.789497	−0.02666	0.076*	
H10C	0.834626	0.718248	0.062297	0.076*	
N1	0.7077 (2)	0.9636 (2)	0.26832 (16)	0.0263 (4)	
H1	0.725256	1.058913	0.272455	0.032*	
O1	0.73962 (17)	0.97953 (18)	0.11063 (13)	0.0272 (4)	
O2	0.69111 (17)	0.75519 (18)	0.16743 (14)	0.0289 (4)	
I1	0.04221 (2)	1.09875 (2)	0.33737 (2)	0.03907 (7)	

### tert-Butyl (5-iodopenta-2,4-diyn-1-yl)carbamate (IV) Atomic displacement parameters (Å^2^)

**Table:**

	*U*^11^	*U*^22^	*U*^33^	*U*^12^	*U*^13^	*U*^23^
C1	0.0294 (14)	0.0398 (14)	0.0301 (13)	0.0020 (11)	0.0106 (10)	0.0027 (11)
C2	0.0333 (14)	0.0328 (13)	0.0263 (12)	−0.0012 (11)	0.0110 (10)	0.0031 (10)
C3	0.0312 (14)	0.0309 (13)	0.0250 (11)	−0.0015 (10)	0.0139 (10)	0.0011 (10)
C4	0.0337 (14)	0.0284 (12)	0.0236 (11)	−0.0036 (10)	0.0135 (10)	0.0005 (9)
C5	0.0298 (13)	0.0321 (13)	0.0259 (12)	0.0007 (10)	0.0132 (10)	0.0032 (10)
C6	0.0180 (11)	0.0216 (11)	0.0271 (11)	0.0013 (8)	0.0087 (9)	0.0020 (9)
C7	0.0490 (17)	0.0323 (13)	0.0293 (13)	−0.0017 (12)	0.0229 (12)	−0.0034 (10)
C8	0.085 (3)	0.0407 (17)	0.0437 (17)	−0.0055 (16)	0.0430 (18)	0.0016 (13)
C9	0.067 (2)	0.065 (2)	0.0325 (15)	−0.0141 (18)	0.0106 (15)	−0.0046 (15)
C10	0.065 (2)	0.0415 (17)	0.063 (2)	0.0074 (15)	0.0446 (18)	−0.0051 (15)
N1	0.0334 (11)	0.0215 (10)	0.0304 (10)	−0.0039 (8)	0.0191 (9)	−0.0015 (8)
O1	0.0396 (10)	0.0196 (8)	0.0293 (9)	0.0004 (7)	0.0209 (7)	0.0004 (7)
O2	0.0348 (10)	0.0197 (8)	0.0352 (9)	−0.0030 (7)	0.0157 (8)	−0.0001 (7)
I1	0.02499 (10)	0.04928 (12)	0.04157 (11)	0.00657 (8)	0.00878 (7)	0.00614 (8)

### tert-Butyl (5-iodopenta-2,4-diyn-1-yl)carbamate (IV) Geometric parameters (Å, º)

**Table:**

C1—C2	1.193 (4)	C7—C9	1.514 (4)
C1—I1	1.999 (3)	C7—C8	1.521 (4)
C2—C3	1.385 (4)	C8—H8A	0.98
C3—C4	1.191 (4)	C8—H8B	0.98
C4—C5	1.472 (3)	C8—H8C	0.98
C5—N1	1.452 (3)	C9—H9A	0.98
C5—H5A	0.99	C9—H9B	0.98
C5—H5B	0.99	C9—H9C	0.98
C6—O2	1.223 (3)	C10—H10A	0.98
C6—O1	1.338 (3)	C10—H10B	0.98
C6—N1	1.340 (3)	C10—H10C	0.98
C7—O1	1.486 (3)	N1—H1	0.88
C7—C10	1.512 (4)		
			
C2—C1—I1	177.3 (3)	H8A—C8—H8B	109.5
C1—C2—C3	179.1 (3)	C7—C8—H8C	109.5
C4—C3—C2	179.4 (3)	H8A—C8—H8C	109.5
C3—C4—C5	177.4 (3)	H8B—C8—H8C	109.5
N1—C5—C4	112.5 (2)	C7—C9—H9A	109.5
N1—C5—H5A	109.1	C7—C9—H9B	109.5
C4—C5—H5A	109.1	H9A—C9—H9B	109.5
N1—C5—H5B	109.1	C7—C9—H9C	109.5
C4—C5—H5B	109.1	H9A—C9—H9C	109.5
H5A—C5—H5B	107.8	H9B—C9—H9C	109.5
O2—C6—O1	125.7 (2)	C7—C10—H10A	109.5
O2—C6—N1	124.3 (2)	C7—C10—H10B	109.5
O1—C6—N1	110.00 (19)	H10A—C10—H10B	109.5
O1—C7—C10	109.6 (2)	C7—C10—H10C	109.5
O1—C7—C9	109.6 (2)	H10A—C10—H10C	109.5
C10—C7—C9	113.4 (3)	H10B—C10—H10C	109.5
O1—C7—C8	101.7 (2)	C6—N1—C5	122.3 (2)
C10—C7—C8	110.5 (3)	C6—N1—H1	118.8
C9—C7—C8	111.5 (3)	C5—N1—H1	118.8
C7—C8—H8A	109.5	C6—O1—C7	121.17 (18)
C7—C8—H8B	109.5		
			
O2—C6—N1—C5	−1.7 (4)	N1—C6—O1—C7	175.9 (2)
O1—C6—N1—C5	178.5 (2)	C10—C7—O1—C6	−58.9 (3)
C4—C5—N1—C6	−103.9 (3)	C9—C7—O1—C6	66.1 (3)
O2—C6—O1—C7	−3.9 (4)	C8—C7—O1—C6	−175.8 (2)

### tert-Butyl (5-iodopenta-2,4-diyn-1-yl)carbamate (IV) Hydrogen-bond geometry (Å, º)

**Table:**

*D*—H···*A*	*D*—H	H···*A*	*D*···*A*	*D*—H···*A*
N1—H1···O2^i^	0.88	2.04	2.881	160
C1—I1···O2^ii^	2.00	2.95	4.919	168

Symmetry codes: (i) −*x*+3/2, *y*+3/2, −*z*+3/2; (ii) −*x*+1/2, *y*+3/2, −*z*+3/2.
